# Event‐Related Potential Components and the Lexical Decision Task in Greek‐Speaking Children With and Without Reading Difficulties

**DOI:** 10.1002/dys.70043

**Published:** 2026-09-08

**Authors:** A. Fella, C. Christoforou, T. C. Papadopoulos

**Affiliations:** ^1^ School of Education University of Nicosia Nicosia Cyprus; ^2^ Division of Computer Science, Mathematics & Science St. John's University New York New York USA; ^3^ Department of Psychology & Centre for Applied Neuroscience University of Cyprus Nicosia Cyprus

## Abstract

The present study examined neurocognitive differences in visual word processing between children with reading difficulties (RD) and chronological‐age‐matched controls (CA) using event‐related potentials (ERPs) during a lexical decision task. Participants were Grade 3 and Grade 6 children (ages 7.6–12.1 years) who were required to discriminate real words from orthographically similar pseudowords. ERP analyses focused on the N200 and N400 components, which are thought to reflect early stages of visual word processing and later processes involved in lexical access and stimulus evaluation. The results showed that the Grade 3 RD group exhibited significantly higher Ν200 amplitudes than their CA controls, suggesting less efficient early processing of the presented stimuli. However, no differences in the N200 were observed between the Grade 6 groups. Children with RD in both grades exhibited reduced N400 amplitudes compared to their counterparts, suggesting differences in the neural processes supporting word recognition. These findings contribute to a clearer understanding of the temporal dynamics of written word processing in children with RD in transparent orthographies and highlight the value of ERP measures for examining neurocognitive mechanisms underlying reading development.

## 1 Introduction

Literacy skills are crucial for academic success and effective daily task management (Alves et al. 2020). As students learn to read and write, they develop the ability to convert spoken language into written form and to decode written words into spoken language to grasp meaning (Papadopoulos et al. 2021). While reading and spelling involve complex cognitive processes (Zoccolotti et al. 2020), most children develop these abilities successfully, without significant difficulty (Hasko et al. 2013). However, some students, particularly those with reading difficulties (RD), face considerable challenges in acquiring literacy skills throughout their school years (Vaughn and Fletcher 2021). Children with RD often struggle to accurately recognise words and achieve fluent word recognition, which can lead to poor comprehension and spelling deficits (e.g., Moll and Landerl 2009; Papadopoulos et al. 2020). This occurs even when their general intelligence, instruction, motivation and sensory or neurological abilities are relatively intact (see APA 2013). Additionally, research has consistently shown that children with RD experience difficulties in various linguistic and cognitive skills, including phonological awareness (Melby‐Lervåg et al. 2012, for a meta‐analysis), rapid automatized naming (Landerl et al. 2019), information processing (T. C. Papadopoulos 2013), working memory (Berninger and Swanson 2014) or visual‐attention processing (e.g., Valdois 2022). Nevertheless, the neural mechanisms underlying these behavioural effects require further investigation, primarily through techniques that can validate findings from behavioural and cognitive assessments.

Studies like the above have primarily focused on cognitive measures tapping reading and reading‐related abilities, with an emphasis on reaction or response times (e.g., Georgiou et al. 2013). However, these measurements capture only a moment at the output stage and overlook the internal cognitive processes involved in actual information processing (e.g., Christoforou et al. 2021). In contrast, Event‐Related Potentials (ERPs)—voltage changes in the ongoing electroencephalogram synchronised with specific internal or external events (Luck 2005, 2023)—provide valuable information beyond accuracy and response time. Due to their exceptional temporal resolution, ERPs provide a detailed account of neural processes as they unfold millisecond by millisecond, filling in gaps often left by behavioural and cognitive assessments (see Kappenman and Luck 2012).

ERPs can reflect the temporal unfolding of multiple processes during reading (Perfetti and Helder 2022). Two ERP components related to reading are the N200 and the N400 (e.g., Breznitz 2003; Fella et al. 2022; Kutas and Federmeier 2011). The N200 is a negative frontocentral deflection peaking around 200 ms after stimulus onset. It is associated with conflict detection, stimulus processing, low‐level linguistic analysis, early visual‐orthographic processing and the manipulation of (visual) material in working memory (Groppe et al. 2011; Karanasiou et al. 2010; López Zunini et al. 2016; Sokhadze et al. 2017). At this stage, the brain begins to differentiate orthographic from non‐orthographic stimuli, often showing stronger neural responses to letter‐like forms, a pattern that has been linked to early visual‐orthographic processing (e.g., Hasko et al. 2013). Consistent with this, the N200 has been linked to access to pre‐lexical orthographic information or to the mapping of pre‐lexical representations onto whole‐word form representations (e.g., Grainger and Holcomb 2009; Liu and Zhang 2023; Welcome and Trammel 2019). At a later stage, meaning retrieval and early integration processes are typically observed in the 300–500 ms time window, which encompasses the N400 component (Perfetti and Helder 2022). The N400 is a negative‐going wave occurring in the centro‐parietal region around 400 ms after stimulus presentation and is commonly associated with later stages of word recognition, lexical access and lexico‐semantic processing (Grainger and Holcomb 2009; Kutas and Federmeier 2011; Welcome and Trammel 2019). Accordingly, the N400 has been widely used to investigate the neural basis of phonological and semantic processing (for review, see Basma, Savage, and Bertone 2024; Kouki, Papadopoulos, et al. 2025; Kutas and Federmeier 2011).

Several studies report atypical N200 responses in individuals with RD, with mixed results (see Clement‐Lam et al. 2026). For instance, Céspedes et al. (2023) reported that children with RD (ages 7–12) exhibited deeper N200 deflections than typically developing controls in response to both words and pseudowords during the lexical decision task. By contrast, Kast et al. (2010) found that typical readers aged 8–12 exhibited larger amplitudes of negative‐going waveforms around 220 ms after stimulus onset during a lexical decision task than RD children. The reduced amplitude observed in the RD group was associated with impaired letter recognition at an early stage of reading processing (Kast et al. 2010).

Differences in later processing stages are also well documented (e.g., Fella et al. 2022; Kouki, Spanoudis, et al. 2025; Silva et al. 2022). In a recent study, Basma, Savage, Luk, and Bertone (2024) calculated the effect size of the N400 component across 21 studies to evaluate reading‐related task performance by individuals with and without RD, reporting an overall effect size of *g* = 0.66. This finding underscores the significance of the N400 in differentiating between typically developing readers and those with RD. Furthermore, Basma, Savage, Luk, and Bertone (2024) noted that altered N400 amplitudes in lexico‐semantic tasks may reflect multiple sources of difficulty rather than a single underlying deficit, including core grapho‐phonological deficits, challenges in processing phonological, and impairments in semantic access, lexical‐semantic interpretation or orthographic conversion processes. Consistent with this, Fella et al. (2022) found that children with RD exhibited prolonged N400 responses during a phonological awareness task, suggesting that Grade 3 children with RD require more time to process phonological stimuli than their peers.

The use of ERPs has advanced our understanding of the reading profiles of children with and without reading difficulties. However, there are two significant limitations in the current research. First, most studies focusing on ERPs in reading tasks have primarily involved participants whose first language was English (e.g., Desroches et al. 2013). This features the need for research involving populations learning to read in more transparent orthographic systems, such as Greek.^1^ Behavioural studies (e.g., Landerl and Wimmer 2008; Papadopoulos et al. 2012) consistently demonstrate that conclusions drawn from English should also be tested in languages with transparent orthographies, as generalising findings from English to other languages can be complex (see Papadopoulos et al. 2021; Share 2008). Second, many relevant studies have involved young adults with a history of RD (e.g., Breznitz 2003; Silva et al. 2022), indicating a need to verify these conclusions in developmental populations. The present study addresses these gaps by examining Greek‐speaking children at two educational stages: Grade 3 (mean age ≈ 8 years) and Grade 6 (mean age ≈ 11 years). These grades correspond to critical phases of reading development in the Greek educational system, marking the transition from basic decoding to fluent, automatized reading (e.g., Fella et al. 2023). To our knowledge, this study is the first to compare differences in the elision of the N200 and N400 components (i.e., peak, mean amplitude and latency) during a lexical decision task between Greek‐speaking participants with reading difficulties and their age‐matched controls. The study involves group comparisons across ages, specifically at 8 (Grade 3) and 11 years (Grade 6). We hypothesise that children with RD will exhibit different N200 and N400 responses in the lexical decision task compared with their age‐matched controls. These differences are expected to be more pronounced in Grade 3, reflecting ongoing development of the neurocognitive processes that support visual word recognition, including phonological, orthographic and semantic processing. In contrast, partial normalisation may be observed by Grade 6 as reading becomes more automatized (e.g., Fella et al. 2022).

## 2 Methods

### 2.1 Participants

Sixty children (36 males and 24 females) participated in the study. The sample included 30 Grade 3 children (M = 8.26 years, SD = 0.60) and 30 Grade 6 children (M = 11.24 years, SD = 0.71). These participants were drawn from 15 urban and rural schools in Cyprus. All schools adhered to the reading curriculum provided by the Ministry of Education, Sport and Youth. The children were native Greek speakers with no reported cognitive, attentional, sensory or behavioural difficulties. Children receiving speech and language therapy were excluded from the study to avoid confounding factors related to reading difficulties. The participants attended public elementary schools in areas of average socioeconomic status, although data on the parents' educational levels and occupations were not provided.

Within the Cypriot educational system, language textbooks are developed by the Greek Ministry of Education, and instruction is conducted in Modern Greek. In primary education, language instruction aims to support students' independent and effective use of language as a structured system of communication, meaning and values. Core instructional objectives include the functional use of grammatical structures, the identification and use of diverse text genres and the comprehension, production, and critical evaluation of texts across written, oral, digital and visual modalities in varied sociocultural settings (Ministry of Education, Culture, Sport and Youth 2022).

The sample was divided into four groups (Grade 3 and Grade 6 children with RD and their CA controls) based on a stepwise selection process, as described below (Table 1).

**TABLE 1:** Descriptive data.

	Gr 3 RD	Gr 3 CA	*x* ^2^/*t*‐test	Gr 6 RD	Gr 6 CA	*x* ^2^/*t*‐test
Gender			0.14			1.29
Boys	8	9		11	8	
Girls	7	6		4	7	
Age						
Years	8.30 (0.27)	8.36 (0.42)	0.32	11.01 (0.92)	11.47 (0.32)	−1.87

*Note:* All *ps* = ns.

Abbreviations: CA: chronological‐age matched group; Gr: grade; RD: group with reading difficulties.

#### 2.1.1 Step I for Group Selection

Because a formal diagnosis of specific learning disability with reading impairment (commonly referred to as reading difficulties) is uncommon in Cyprus, teachers were asked to nominate students from their classrooms who had difficulty decoding words at an age‐appropriate rate, provided there were no sensory, intellectual or attention‐related issues. Research has shown that teachers' assessments of students' reading levels are usually confirmed by the students' subsequent reading scores (Virinkoski et al. 2018). Teachers independently evaluated the nominated students using a 12‐item reading ability checklist, scored on a Likert scale from 1 (does not apply) to 4 (definitely applies). Students who received ratings below the 20th percentile on the reading ability scale were selected for the RD groups. The reliability of this scale, as indicated by Cronbach's alpha, is 0.90 (see Spanoudis et al. 2019).

#### 2.1.2 Step II for Group Selection

After obtaining parental consent, the selected children were evaluated on reading fluency and general cognitive ability to ensure they met the criteria for reading difficulties, consistent with commonly used definitions of specific learning disability with impairment in reading (American Psychiatric Association 2013). Reading fluency tasks were used to assess the reading rate, as children with RDs in Greek typically perform well in accuracy. Greek is a transparent language with high consistency between graphemes and phonemes (T. C. Papadopoulos 2001). The study included 15 Grade 3 children (8 boys, 7 girls; mean age = 8.30 years, SD = 0.27) and 15 Grade 6 children (11 boys, 4 girls; mean age = 11.01 years, SD = 0.92). These children scored at least one standard deviation below their age‐group mean on the reading fluency tasks (word reading fluency and phonemic decoding fluency; ERS‐AB; Papadopoulos, Spanoudis, and Kendeou 2009) and within the average range on verbal (assessed using the Vocabulary Wechsler Intelligence Scale for Children, Wechsler 2014·Greek standardisation: Stogiannidou 2017) and non‐verbal ability tasks (assessed using Nonverbal Matrices from the DN‐CAS, Naglieri and Das 1997; Greek standardisation: Papadopoulos, Georgiou, et al. 2009).

#### 2.1.3 Step III for Group Selection

A separate group of 15 Grade 3 children (9 boys, 6 girls; mean age = 8.36 years, SD = 0.42) and 15 Grade 6 children (8 boys, 7 girls; mean age = 11.47 years, SD = 0.32) with age‐appropriate reading rates were randomly selected from the same classes and matched to the RD groups based on both chronological age and gender. No significant differences were observed between the RD and control groups on verbal and non‐verbal measures, Wilks' *L* = 0.98, *F*(2, 57) = 0.70, ns.

### 2.2 Lexical Decision Task

A computerised version of the lexical decision task was adapted from Papadopoulos, Spanoudis, and Kendeou (2009). Each trial began with a fixation cross displayed at the centre of a computer screen for 1500 ms. This was followed by presenting the first letter string for 2000 ms, after which a second fixation cross was shown for 1500 ms. The second letter string was then displayed for 2000 ms, followed again by a third fixation cross. After a 1500‐ms interval, both letter strings were presented simultaneously on the screen. Participants were instructed to identify the real word from a pseudoword containing similar letters by pressing the designated keys on the keyboard. This sequential presentation prior to the decision phase was implemented to standardise exposure to both stimuli and to reduce reliance on immediate visual salience during the final comparison, a factor known to influence lexical decision performance (e.g., Balota and Chumbley 1984). The paradigm was therefore designed to emphasise discrimination between orthographically similar stimuli rather than rapid lexical classification. Specifically, participants were instructed to press the ‘a’ button on the keyboard with the index finger of their left hand when the real word appeared on their left and the ‘l’ button with the index finger of their right hand when the real word appeared on their right. Colour stickers were used to prevent confusion with the response buttons, with red colour for the ‘a’ button and blue colour for the ‘l’ button. Following the participants' response, a new trial commenced after an inter‐trial interval of 2000 ms.

**FIGURE 1:**
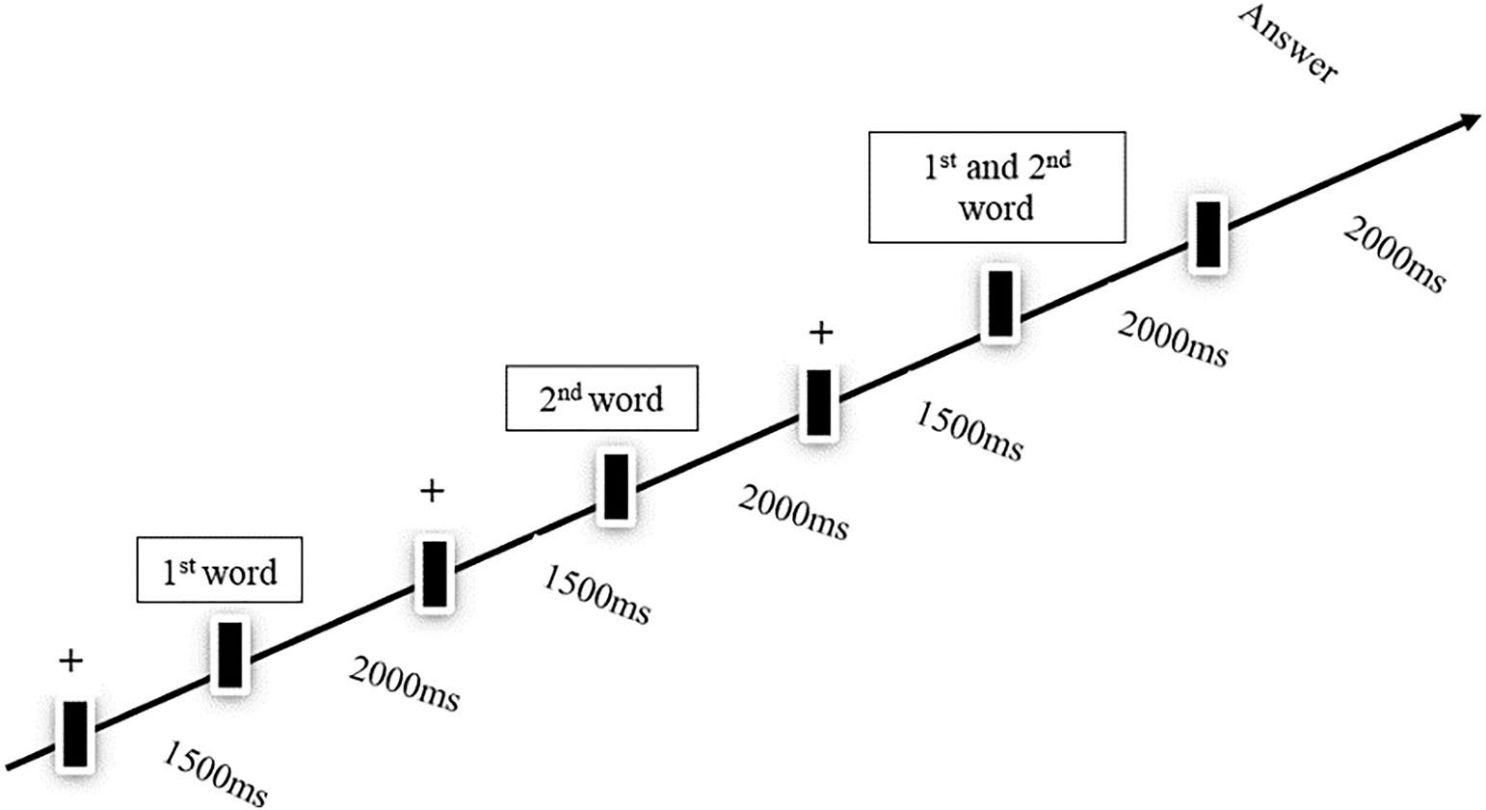
Sample stimuli of the lexical decision task.

The lexical decision task consisted of 240 word pairs of 120 high‐frequency and 120 low‐frequency words, presented in the same order for all participants. For each word, three different types of pseudowords were created by (a) replacing an interior letter from the corresponding real word, (b) omitting a letter from the real word and (c) reversing the order of two consecutive letters within the real word (see Protopapas et al. 2013). Across trials, the type of pseudoword paired with each real word varied, ensuring that all three pseudoword types were presented in a non‐systematic order. For example, the pseudowords /kilkoforiakis/, /kiloforiakis/ and /kokloforianis/ were created for the word /kikloforiakis/. To control for potential order effects, the sequence of stimulus presentation within each pair was counterbalanced. In half of the trials, the real word was presented first, followed by the pseudoword during the sequential presentation phase, whereas in the remaining trials, the pseudoword was presented first, followed by the real word. The words used in the study were sourced from school‐language textbooks, ensuring they fell within the participants' broader vocabulary range. The frequency of each specific word was determined based on its occurrence in language textbooks from Grade 1 to Grade 6 (see Papadopoulos and Loizou 2007). The stimulus length ranged from two to seven syllables. Children were given a short break (1 min) after every 40 trials to mitigate potential fatigue. All words and pseudowords were presented in black lowercase letters on a white background. The stimuli were presented on a Dell Precision T5500 workstation with an ASUS VG‐236 monitor (1920 × 1080, 120 Hz, 52 × 29 ch) at a viewing distance of 60 cm. The experiment was programmed and presented using Experiment Builder (SR Research Ltd 2016). A preliminary testing phase was conducted, which included five pairs of words (Figure 1).

### 2.3 Procedure

Participants underwent individual testing sessions lasting approximately 50 min. The order of task administration was consistent across all participants. Initially, tasks assessing reading fluency, verbal ability and non‐verbal ability were administered, followed by the lexical decision task. The sessions took place in a quiet room at the Center for Applied Neuroscience, University of Cyprus, during extracurricular hours, such as weekends. Written consent was obtained from both schools and parents prior to testing. The study received approval from the Ministry of Education, Sport, and Youth in Cyprus (approval number #7.15.01.27/17) and was conducted in accordance with the guidelines set forth by the Cyprus National Bioethics Committee (EEBK/EP/2011/10).

### 2.4 Event Related Potentials and Data Manipulation

EEG data were collected via active electrodes positioned at 64 scalp sites (Fp1, Fpz, Fp2, AF7, AF3, AFz, AF4, AF8, F7, F5, F3, F1, Fz, F2, F4, F6, F8, Ft7, Fc5, Fc3, Fc1, Fcz, Fc2, Fc4, Fc6, Ft8, T7, C5, C3, C1, Cz, C2, C4, C6, T8, Tp7, Cp5, Cp3, Cp1, Cpz, Cp2, Cp4, Cp6, Tp8, P9, P7, P5, P3, P1, Pz, P2, P4, P6, P8, P10, PO7, PO5, POz, PO4, PO8, O1, Oz, O2 and Iz), according to the 10/10 system. The sampling rate was set at 512 Hz, and data collection was continuous throughout the experiment. The DC offset of all sensors was monitored using the BioSemi Active‐Two system (BioSemi, Amsterdam, Netherlands) and was maintained below 20 μV with electro‐gel.

EEG data pre‐processing was performed offline using custom MATLAB scripts (http://www.mathworks.com) and the EEGLAB library. Initially, the EEG data were downsampled to 256 Hz, and all channels were re‐referenced to the average channel. A high‐pass filter with a cut‐off frequency of 0.5 Hz was applied to the continuous EEG signal to eliminate DC drifts. This was followed by a 50 Hz notch filter to reduce the power‐line noise interference. Subsequently, a 25 Hz low‐pass filter was used to minimise noise from high‐frequency components irrelevant to ERP analysis.

Next, the continuous EEG signal was segmented into epochs ranging from 200 ms before to 600 ms after the onset of each event (e.g., first letter string). The baseline amplitude (from −200 to 0 ms of the event onset) was removed from each epoch. Trials containing artefacts were automatically removed using the ‘pop_rejchan’ and ‘pop_autorej’ functions from the EEGLAB library, followed by a verification step to ensure that at least 200 trials per subject were kept (see Christoforou et al. 2013). Finally, ERPs for each participant were calculated by averaging across all the corresponding epochs. The latencies and amplitudes (mean and peak) of the N200 and N400 components were calculated across six distinct channel groups (see Table 2).

**TABLE 2:** Selected channels during the lexical decision task.

Group	Channels
Lexical decision task	
1.	F1, Fz, F2 (frontal regions)
2.	C1, Cz, C2 (central regions)
3.	FC1, FCz, FC2 (frontocentral regions)
4.	F7, F8 (frontal regions)
5.	Fp1, AF3, F1, Fpz, Fp2, AFz, Fz, F2 (frontal regions)
6.	FT7, FC5, FC6, FT8 (frontal regions)

For statistical analysis, the N200 component was quantified as mean and peak amplitude within a 130–230 ms time window, and the N400 component within a 300–450 ms time window, relative to stimulus onset. These windows were selected based on visual inspection of the grand‐average waveforms across groups and channels, as well as on typical latencies reported in the ERP literature on word recognition and reading (e.g., Grainger and Holcomb 2009; Hasko et al. 2013; Kutas and Federmeier 2011). The chosen time windows are illustrated in Figures S1 and S2, where shaded regions indicate the intervals used for N200 and N400 quantification. The selection of the specific channel groups was guided by prior work identifying central electrodes, particularly those surrounding Cz, as key sites for capturing components relevant to reading (e.g., Breznitz 2002; Fella et al. 2022). In line with this literature, we focused on central, fronto‐central and fronto‐temporal regions. Within these broader ROIs, channels were grouped based on spatial proximity. Visual inspection of the grand‐average waveforms confirmed similar response patterns within each channel group (Table 2). This approach allowed us to anchor our ROI selection in both established findings and in the observed similarity patterns across channels. Grant average ERPs for children with and without RD were computed by averaging across the ERPs of all children in each group.

## 3 Results

### 3.1 Behavioural Performance

A multi‐variate analysis of variance (MANOVA) was conducted, with descriptive variables (accuracy and time) in the lexical decision task as dependent measures and group (4) as a fixed factor. The multivariate effect of group was significant (Wilks' *L* = 0.398, *F*(6,110) = 10.72, *p* < 0.001, *η* ^2^ = 0.37). Follow‐up univariate analyses revealed significant main effects of group for both accuracy (*F*(3, 56) = 22.62, *p* < 0.001, *η* ^2^ = 0.55) and response time (*F*(3, 56) = 10.88, *p* < 0.001, *η* ^2^ = 0.37). Post hoc comparisons using Bonferroni correction and effect size estimates (Cohen's d) indicated significant differences between the children with RD and the control groups. Specifically, the Grade 3 RD group performed significantly less accurately than the CA‐matched group on the lexical decision task (*d* = 1.40). In addition, the Grade 6 RD group was significantly slower (*d* = 0.87) and less accurate (*d* = 1.49) than the CA‐matched group (Table 3). These analyses were based on overall task performance, collapsed across word‐length and frequency conditions.

**TABLE 3:** Behavioural data.

	Gr3 RD	Gr3 CA	*d* ^1–2^	Gr6 RD	Gr6 CA	*d* ^3–4^
	Μ (SD)	Μ (SD)		Μ (SD)	Μ (SD)	
Lexical decision task (overall)						
Accuracy	185.07 (27.58)^2,3,4^	215.27 (13.11)^4^	1.40	220.00 (11.72)	232.47 (1.73)	1.49
Speed	214.27 (43.16)^3,4^	191.21 (41.11)^4^	0.55	167.29 (47.80)	131.52 (32.55)	0.87
Accuracy by word frequency and length						
HF, 2–3 syllables	12.47 (1.85)^2^	16.60 (2.87)	1.71	16.73 (3.53)	19.07 (1.98)	0.82
HF, 4–5 syllables	10.40 (3.42)^2^	15.87 (2.90)	1.72	15.80 (3.55)	17.87 (1.73)	0.74
HF, 6+ syllables	4.80 (1.93)	6.00 (1.96)	0.62	6.87 (1.85)^4^	8.80 (1.26)	1.22
LF, 2–3 syllables	10.80 (3.14)^2^	13.87 (3.38)	0.94	14.80 (2.48)	17.00 (2.75)	0.84
LF, 4–5 syllables	9.13 (2.67)^2^	11.33 (2.06)	0.92	13.13 (2.83)	15.07 (2.02)	0.78
LF, 6+ syllables	4.33 (1.63)	5.20 (1.86)	0.50	6.33 (1.63)^4^	7.73 (1.67)	0.85

*Note:* Accuracy values in the word‐type analyses represent the mean number of correctly identified items per condition.

Abbreviations: CA: chronological‐age matched group; *d*:Cohen's d; Gr: grade; HF: high frequency; LF: low frequency; M: mean; RD: group with reading difficulties; SD: standard deviation.

The superscripts indicate statistically significant differences between groups; differences between groups are marked only from left to right: ^1^Group 1 (Gr3 RD); ^2^Group 2 (Gr3 CA); ^3^Group 3 (GR6 RD); ^4^Group 4 (Gr6 CA).

To further examine whether behavioural performance varied as a function of word characteristics, additional analyses were conducted separately for word‐length and word‐frequency conditions (see Table 3). In Grade 3, children with RD performed significantly less accurately than their CA controls on high‐frequency short words (*d* = 1.71, *p* < 0.001), high‐frequency medium‐length words (*d* = 1.72, *p* < 0.001), low‐frequency short words (*d* = 0.94, *p* = 0.016) and low‐frequency medium‐length words (*d* = 0.92, *p* = 0.017). In Grade 6, group differences were most pronounced for long words, with children with RD performing less accurately than controls for both high‐frequency (*d* = 1.22, *p* = 0.002) and low‐frequency items (*d* = 0.85, *p* = 0.028).

### 3.2 ERP Data

A series of multi‐variate ANOVAs was conducted with group (4) as a fixed factor and peak amplitude, mean amplitude and peak latency for the N200 and N400 components as dependent measures. These analyses were performed across six different predefined channel groups. Additional analyses were performed based on (a) *word status* (real words vs. pseudowords, regardless of length and frequency), (b) *word frequency* (high vs. low frequency, regardless of length) and (c) *word length* [short words (2–3 syllables) vs. medium words (4–5 syllables) vs. long words (6+ syllables), regardless of frequency]. The analytical strategy was based on specific hypotheses. ERP components and channel groups were selected in advance, guided by previous studies and the scalp distribution of the grand‐average waveforms (e.g., Breznitz 2002; Fella et al. 2022). Each channel group, waveform and dependent measure was analysed separately to capture the temporal and spatial location of the ERP responses. To reduce the risk for false positives from multiple comparisons, Bonferroni correction was applied within each set of statistical tests and only effects that stayed significant after correction were interpreted.

### 3.3 N200 Component

**FIGURE 2:**
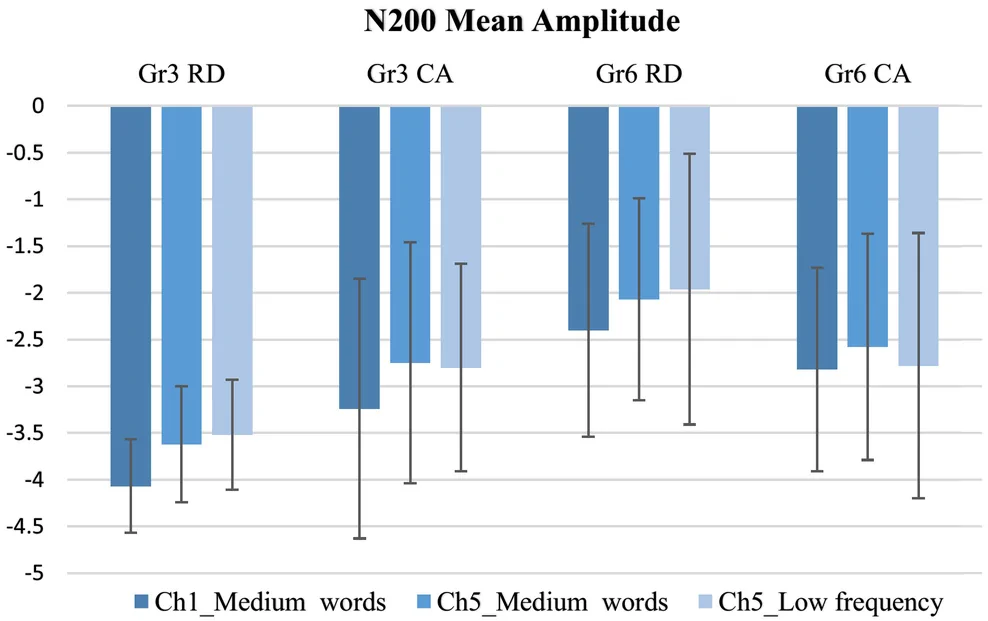
Group differences in N200 mean amplitude for Grade 3 and Grade 6 participants. CA: chronological‐age matched group; Ch: channel; RD: group with reading difficulties.

For the N200 *mean amplitude*, the multivariate test revealed significant effects in the first (Wilks' *L* = 0.488, *F*(21,144) = 0.95, *p* < 0.05, *η* ^2^ = 0.21) and a fifth group of channels (Wilks' *L* = 0.544, *F*(21,144) = 1.62, *p* = 0.05, *η* ^2^ = 0.18), both located in the frontal brain regions. Subsequent univariate tests showed significant effects for medium‐length words in both channel groups (*F*(3, 56) = 6.54, *p* = 0.001, *η* ^2^ = 0.26 and *F*(3, 56) = 5.34, *p* < 0.01, *η* ^2^ = 0.22, respectively), regardless of frequency. In the fifth group of channels, significant effects were also found for low‐frequency words (*F*(3, 56) = 4.33, *p* < 0.01, *η* ^2^ = 0.19), regardless of length. Effect size comparisons (Cohen's d^2^) showed that the mean amplitude of the N200 component in the Grade 3 RD group was significantly larger than that of the CA controls for both medium (*d* = 0.79 and *d* = 0.86 in the first and fifth channel groups, respectively) and low‐frequency words (*d* = 0.81) (Figure 2).

**FIGURE 3:**
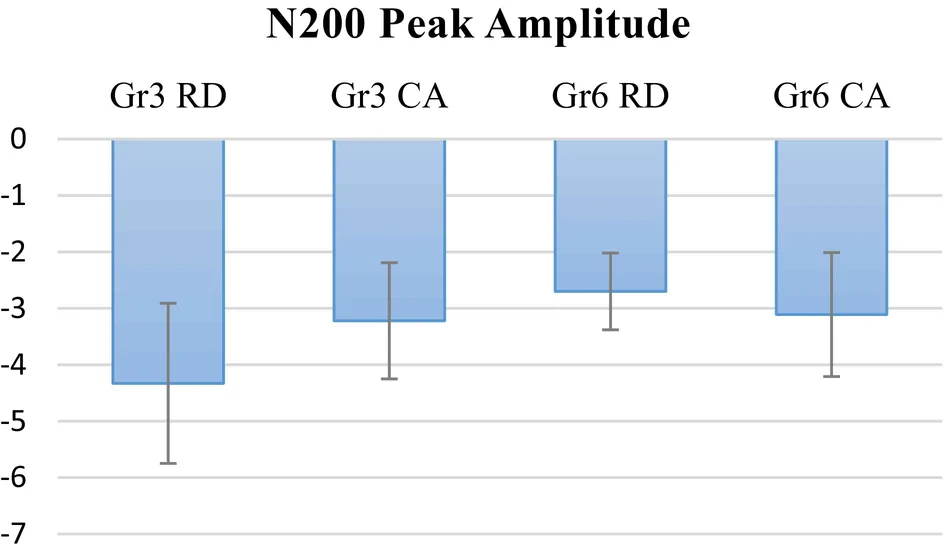
Group differences in N200 peak amplitude for Grade 3 and Grade 6 participants. CA: chronological‐age matched group; RD: group with reading difficulties.

For the N200 *peak amplitude*, significant main group effects were observed in the sixth group of channels (Wilks' *L* = 0.627, *F*(9, 131) = 3.09, *p* < 0.01, *η* ^2^ = 0.14) over the frontal regions. Subsequent univariate analyses showed that the main effect on the group was significant for long words, regardless of frequency (*F*(3, 56) = 6.17, *p* = 0.001, *η* ^2^ = 0.25). Effect size comparisons indicated that the Grade 3 RD group exhibited significantly enhanced N200 peak amplitude compared to controls when processing long words (*d* = 0.89) (Figure 3).

The *latency* of the N200 component was similar across Grade 3 groups. Additionally, no significant differences in the N200 component were observed between the Grade 6 groups. Overall, the significant N200 effects were restricted to frontal and fronto‐central channel groups (Groups 1, 5 and 6), with no systematic differences between left‐ and right‐hemisphere electrodes within these regions, suggesting a region‐specific rather than a clear hemispheric laterality effect.

### 3.4 N400 Component

**FIGURE 4:**
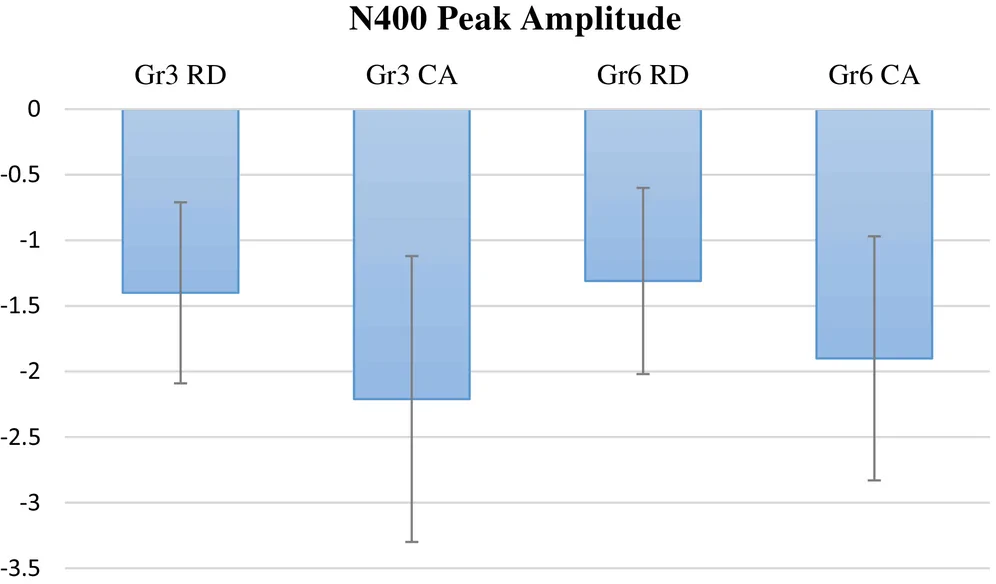
Group differences in N400 peak amplitude for Grade 3 and Grade 6 participants. CA: chronological‐age matched group; RD: group with reading difficulties.

For the N400 component, the analysis showed a significant multivariate effect of group on *peak amplitude* in the fourth group of channels (Wilks' *L* = 0.736, *F*(9, 131) = 1.96, *p* < 0.05, *η* ^2^ = 0.10), located in the frontal regions of the brain. Follow‐up univariate analyses revealed significant group effects for long words, regardless of frequency (*F*(3, 56) = 2.85, *p* < 0.05, *η* ^2^ = 0.13). Effect size comparisons indicated that children with RD in both Grades 3 and 6 presented reduced N400 amplitudes compared to controls when processing long words (*d* = 0.89 and *d* = 0.71, respectively). No significant differences in N400 mean amplitude or N400 latency were observed between the Grade 6 groups. For the N400, group differences were likewise confined to frontal electrodes (Group 4), with no robust evidence for hemispheric asymmetries within the predefined regions of interest (Figure 4).

## 4 Discussion

This study examined differences between children with reading difficulties (RD) and their typically developing peers of the same chronological age (CA) on a lexical decision task using Event‐Related Potentials (ERPs). We specifically focused on the N200 and N400 components in Greek‐speaking children. This research is significant because previous studies have primarily focused on adults with developmental RD and their CA controls, all of whom were English‐speaking. As a result, there is limited information on how ERP components help distinguish between children with and without RD in languages with consistent orthographies, such as Greek.

Our results lead to several key conclusions. First, since the amplitude of the N200 reflects task difficulty (Benikos et al. 2013; Senkowski and Herrmann 2002), our findings suggest that the Grade 3 group with RD faces significant challenges with processing medium‐ to long‐length words regardless of frequency, as well as low‐frequency words of any length. The increased N200 amplitude in the Grade 3 RD group likely indicates a greater processing effort in decoding the visual features of the presented items at the early reading stage (Kast et al. 2010). Maurer et al. (2007) have suggested that coarse visual tuning for print develops around 150–270 ms and progresses more slowly in young children with RD than in their typically developing controls. Similarly, Kouki, Papadopoulos, et al. (2025), in their investigation of the neural correlates of print expertise in school‐age children, found that children with RD exhibited attenuated amplitudes in the 150–250 ms time window, a pattern suggestive of weaker phonological representations. This finding is consistent with the view that early cortical processes involved in prelexical orthographic analysis may be less efficient or develop differently in children with RD (Araújo et al. 2012; Maurer et al. 2007). This interpretation is further supported by the behavioural findings. Specifically, Grade 3 children with RD performed less accurately than their CA controls on medium‐length and low‐frequency items, the same stimulus categories that elicited significant N200 differences. Thus, these findings suggest that the observed N200 effects were associated with measurable difficulties in processing more demanding lexical stimuli. However, by Grade 6, children with RD may develop compensatory mechanisms that enable them to use perceptual encoding of orthographic properties in a manner similar to their typically developing peers. At the same time, the behavioural findings indicate that this compensation may remain incomplete, as the Grade 6 group with RD showed lower accuracy and slower response times across the task overall. Therefore, even if older children with RD recruit partially adaptive neural mechanisms, these do not seem sufficient to normalise performance fully.

Second, consistent with previous studies (see Basma, Savage, Luk, and Bertone 2024), our results demonstrated significant group differences in the N400 amplitude. Specifically, RD groups showed reduced N400 amplitudes when processing long words compared to their age‐matched controls. Within the selected channel groups, both the N200 and N400 effects were most evident over frontal and fronto‐central regions. This pattern, however, warrants cautious interpretation because parietal electrodes were not included in the present analysis. As such, we cannot determine whether the observed N400 reflects a genuinely atypical anterior shift relative to the more commonly reported centro‐parietal distribution (e.g., Kutas and Federmeier 2011). Rather, our findings indicate that, within the scalp regions examined here, long‐word processing in students with RD was associated with reduced N400 amplitudes and greater involvement of anterior regions. One possible interpretation is that these effects reflect increased recruitment of controlled, effortful or compensatory processes during difficult word recognition in RD (Kouki, Papadopoulos, et al. 2025; Lau et al. 2008; Schulz et al. 2008, 2009). Within these frontal regions, however, we did not observe robust or systematic hemispheric laterality effects, likely reflecting both the use of aggregated channel groups and the fact that the study was not specifically designed or powered to test fine‐grained left–right asymmetries. This pattern of neurophysiological activity is broadly consistent with previous findings linking N400 differences to less efficient orthographic and lexical processing in children with RD (Basma, Savage, Luk, and Bertone 2024; Hasko et al. 2013). The observed differences in N400 amplitudes suggest that students with RD engage in more effortful processing of long stimuli, highlighting their deficient automaticity. As a result, these students rely heavily on phonological decoding to read long words successfully (Fella and Papadopoulos 2018; Papadopoulos et al. 2014). The behavioural findings also support this interpretation. In Grade 6, the largest differences between children with RD and their CA controls emerged for long words. Notably, long words were also the condition under which reduced N400 amplitudes were observed. Therefore, these findings suggest that the N400 differences were associated with difficulties in processing more complex lexical stimuli. Previous studies have shown that individuals with RD often employ a serial sub‐lexical decoding rather than a parallel lexical reading strategy (Papadopoulos et al. 2021; Shaywitz and Shaywitz 2008). Additionally, these findings are consistent with empirical evidence from eye‐movement studies, which have consistently demonstrated that individuals with RD process letter strings sequentially, one at a time, and benefit less from parafoveal processing than proficient readers (e.g., Fella et al. 2023). This could potentially affect their reading speed and overall reading proficiency.

One possible interpretation is that the reduced N400 effect reflects differences in the engagement of frontal brain mechanisms involved in reading and language processing (Lau et al. 2008; Schulz et al. 2009). Schulz et al. (2008) used counterbalanced sessions with functional magnetic resonance imaging (fMRI) and ERPs. They found that children with RD showed decreased activation in the frontal regions of the language network and the N400 effect during sentence reading. Similarly, Kouki, Spanoudis, et al. (2025), in a study of Greek‐speaking children, found that children with RD and typically developing peers showed different N400 patterns in both amplitude and lateralization during a lexical‐semantic task. Specifically, typical readers exhibited a stronger left‐lateralized N400 response in the implausible condition. In contrast, children with RD showed a stronger right‐lateralized frontal N400 response, suggesting the use of compensatory strategies to offset reduced activation in left frontal regions. However, hemispheric lateralization was not examined in the present study; therefore, our data do not allow conclusions regarding whether similar lateralized compensatory mechanisms were present in our sample. Future studies employing electrophysiological and neuroimaging techniques to investigate N400‐related source activity in transparent orthographies may further support this perspective.

The current findings present several educational and psychological implications, especially for children with RD in orthographically consistent languages. Educators and psychologists must recognise the valuable information ERP data can provide in understanding reading behaviours and individual differences among students. ERP findings can help us better understand the neurocognitive processes underlying reading difficulties and may inform the development and evaluation of intervention methods (e.g., Wilcox et al. 2021). For instance, atypical N400 patterns have been associated with poorer reading performance and may reflect less efficient processing during later stages of word recognition and lexical access. According to the self‐teaching hypothesis, phonological functions can serve as a self‐teaching mechanism, allowing learners to acquire automaticity and develop an independent orthographic lexicon (Share 1995, 2008). Thus, intervention programs that strengthen the cognitive and linguistic processes associated with successful word recognition, particularly phonological skills, may contribute to more efficient neural processing during reading (Christoforou et al. 2023; Lyytinen et al. 2009; Papadopoulos et al. 2004; Yeratziotis et al. 2025).

A few limitations should be noted. First, while these findings are consistent with differences in orthographic and lexical processing between children with and without RD, the ERP components studied here are not linked only to orthographic representations. The N200 and N400 can also be affected by broader cognitive processes, such as attention, working memory, comparison of stimuli and decision‐making. Therefore, these results do not directly show impaired orthographic representations. Instead, they point to differences in the neurocognitive processes involved during the lexical decision task. Second, because the research was conducted in Greek, the extent to which the findings generalise across languages with less transparent orthographies remains uncertain. Third, although participants were identified using established criteria for reading difficulties (e.g., Papadopoulos et al. 2020), different studies employ somewhat different classification thresholds and procedures (Landerl et al. 2013; Sumner et al. 2013). Finally, the lexical decision paradigm was designed to maximise sensitivity to subtle orthographic differences between words and pseudowords; it required participants to encode and compare sequentially presented stimuli before responding. As a result, the task may have recruited additional cognitive processes, including attentional control, working memory and decision‐making mechanisms. Despite these limitations, the present study is among the few studies to examine ERP correlates of lexical processing in children with reading difficulties learning to read in a transparent orthography.

In conclusion, our study revealed that Grade 3 children with reading difficulties exhibited increased N200 amplitudes during a lexical decision task, suggesting less efficient neural processing during the early stages of word recognition. Furthermore, children with RD in both grades exhibited reduced N400 amplitudes, indicating differences in the neurocognitive processes supporting later stages of word recognition. These findings advance our understanding of the temporal dynamics of reading development among children learning to read in a transparent orthography.

## Funding

The dissemination activities were supported by the University of Nicosia (grant awarded to Argyro Fella). The research was partly supported by a Cyprus Research Promotion Foundation grant and the European Regional Development Fund (ERDF) under the EXCELLENCE HUBS/1216/0508 programme (grant awarded to Timothy C. Papadopoulos).

## Conflicts of Interest

Timothy Papadopoulos is an Associate Editor for Dyslexia.

## Supporting information


**Figure S1:** Grand‐average ERP waveforms for differences between Gr3 RD and Gr3 CA/ROI, with shaded areas indicating the 130–230 ms window for the N200 and the 300–450 ms window for the N400 used for statistical analyses.
**Figure S2:** Grand‐average ERP waveforms for differences between Gr6 RD and Gr6 CA/ROI, with shaded areas indicating the 130–230 ms window for the N200 and the 300–450 ms window for the N400 used for statistical analyses.

## Data Availability

The data may be available from the corresponding author upon reasonable request, subject to the relevant permissions and restrictions.

## Endnotes

^1^
Protopapas and Vlachou (2009) reported that the overall consistency for Greek at the grapheme‐phoneme level is 95.1% in the feedforward direction and 80.3% in the feedback direction. On this basis, the authors concluded that Greek is sufficiently transparent. This transparency facilitates early decoding accuracy, enabling Greek‐speaking children to achieve high levels of word‐reading accuracy within the first years of formal schooling. However, despite early mastery of accuracy, difficulties in reading fluency and automaticity often persist, particularly among children with reading difficulties (Papadopoulos et al. 2012).

^2^
Cohen's d is an effect size used to describe the standardised difference between two means. A generally accepted minimum level of power is 0.80 (Cohen 1969).
