# Supplementary material for: Event‐Related Potential Components and the Lexical Decision Task in Greek‐Speaking Children With and Without Reading Difficulties
Source: Dyslexia. 2026 Sep 8;32(4):e70043. doi: 10.1002/dys.70043 (PMC13552215; doi:10.1002/dys.70043)
Supplement: Supplementary file 1 [file DYS-32-e70043-s001.docx]

**Appendix**

**Supplementary Figure 1.** Grand-average ERP waveforms for differences between Gr3 RD and Gr3 CA/ROI, with shaded areas indicating the 130-230 ms window for the N200 and the 300-450 ms window for the N400 used for statistical analyses

**Supplementary Figure 2.** Grand-average ERP waveforms for differences between Gr6 RD and Gr6 CA/ROI, with shaded areas indicating the 130-230 ms window for the N200 and the 300-450 ms window for the N400 used for statistical analyses
